# A novel method of grading gastric intestinal metaplasia based on the combination of subtype and distribution

**DOI:** 10.1186/s12935-021-01758-6

**Published:** 2021-01-20

**Authors:** Ning Wei, Zhiheng Zhong, Ruihua Shi

**Affiliations:** 1 Medical School of Southeast University, No. 87 Dingjiaqiao,, Nanjing, 210009 China; 2 Department of Gastroenterology, Southeast University Affiliated Zhongda Hospital, No. 87 Dingjiaqiao, Nanjing, 210009 China

**Keywords:** Gastric intestinal metaplasia, Subtype, Distribution, The difference between the body and gastric antrum lesions, New evaluation system

## Abstract

**Background:**

Studies have shown the value of subtypes and distribution of gastric intestinal metaplasia (GIM) for prediction of gastric cancer. We aim to combine GIM subtypes and distribution to form a new scoring system for GIM.

**Methods:**

This was a cross-sectional study. No GIM, type I, II, and III GIM of gastric antrum and corpus scored 0–3 points respectively. Then the severity of the whole stomach was calculated in two ways: 1. The gastric antrum and corpus scores were added together, with a score ranging from 0 to 6, which named “Subtype Distribution Score of Gastric Intestinal Metaplasia (SDSGIM)”. 2. Direct classification according to a table corresponding to that of OLGIM. We compared the SDSGIM among benign lesions, dysplasia, and cancer and drew receiver operating characteristic (ROC) curve to determine the optimal cut-off value. According to the cut-off value and the classification from the table, the predictive ability of these two methods were calculated.

**Results:**

227 patients were included. For SDSGIM, benign lesion group was significantly different from dysplasia or cancer group. Area under curve of ROC curve was 0.889 ± 0.023. The optimal cut-off value was 3. The sensitivity, specificity, positive predictive value, negative predictive value, and accuracy of SDSGIM for malignancy were 89.5%, 78.0%, 74.6%, 91.2% and 82.8%. And those for the second classification method were 84.2%, 82.6%, 77.7%, 87.9%, and 83.3% respectively.

**Conclusions:**

This study firstly combined GIM subtypes with its distribution forming a novel scoring system, which showed high prediction accuracy for malignant lesions.

## Background

Development of gastric adenocarcinoma is a complex multistep process described as Correa’s cascade [1], which includes an important pre-malignant stage of gastric mucosal intestinal metaplasia (GIM). The annual incidence of gastric cancer has been reported to be 0.25% in patients diagnosed with GIM [2]. However, there is currently no specific treatment for GIM, so it is very important to identify high-risk patients who will progress to gastric cancer and regularly monitor [3, 4].

Several high gastric cancer risk factors have been identified, including physiological and life-style components. The physiological factors, such as bile reflux, older age, male sex, and body mass index (BMI) were shown to predispose to development of GIM and gastric malignancies. Some of these factors are interconnected. For instance, higher BMI and abdominal fat deposits promote bile reflux by increasing intra-abdominal pressure, thus, increasing probability of intestinal malignancies [5]. The most influential life-style risk factors, smoking and drinking [6], were indicated to aggravate chronic gastritis. Infection with H. pylori, a prolonged adverse exposure factor, is known to increase the severity and distribution of precancerous lesions [7].

The OLGIM (Operative Link on Gastric Intestinal Metaplasia) staging system was also developed [8] to comprehensively assess and score the severity of GIM. According to the proportion of intestinal metaplasia cells under the microscope, the GIM severity of gastric antrum (at least 3 biopsies) and gastric body (at least 2 biopsies) is divided into four levels (none, mild, moderate, and severe (or marked)), and then integrate the severity of GIM of the antrum and gastric body and stratify the GIM according to the OLGIM grading table (Additional file 1: Table S1, Figure S1). However, some study guidelines didn’t recommend to use OLGIM in clinical practice [3, 4] due to the subjectivity of pathological grading and the requirement of multiple biopsies. With the development of chromoendoscopy and digital chromoendoscopy (DC), new diagnostic choices were introduced. However, there are some limitations of this method. Chromoendoscopy requires application of dyeing agents, which is complicated and time-consuming procedure. Therefore, it has been gradually replaced by electronic dyeing endoscopes. In 2016, Pimentel-Nunes Pedro et al. [9] proposed the concept of endoscopic grading of gastric intestinal metaplasia (EGGIM). The method is mainly based on using of the narrow band imaging (NBI) system to score the extent of GIM in the whole stomach. The method is effective and helps to identify high-risk patients. Although this method has strong clinical practicability and allows to observe micro vascularization, it also has disadvantages of being subjective and time-consuming. The method relays on high-resolution DC images and highly trained and experienced physicians. Therefore, a new simple and effective risk stratification scheme is needed to identify high-risk patients for further regular follow-up and careful examination (Additional file 2).

Several studies have shown that patients with GIM in the gastric corpus have higher chances of developing gastric cancer compared to patients with GIM of the gastric antrum [10, 11]. However, controversial data was published about the predictive ability of GIM subtyping for dysplasia/cancer [12–14]. Several study guidelines didn’t recommend using it [3, 4] in the past. But more and more studies [15, 16] including a meta-analysis [17] have shown that type II and III intestinal metaplasia (Subtype of GIM include Type I II and III, and from type I to III the chance of malignant transformation is increasing) may indeed mean a higher risk of gastric cancer in recent years. Both GIM subtypes and distribution in the antrum and body of stomach have a certain predictive value on GIM progression to gastric cancer, although no attempts have been made to combine these two factors and form a new GIM risk stratification system. Therefore, we aim to combine GIM subtypes and distribution to form a new scoring system and evaluate its ability to predict the risk of gastric dysplasia/cancer.

## Methods

### Patients and data collection

This cross-sectional study was carried out at Zhongda Hospital of Southeast University, which was performed based on the Declaration of Helsinki. All patients signed an informed consent before endoscopy. Patients who were hospitalized in our Gastroenterology from June 2019 to October 2020 and was performed with NBI and white light endoscopy by 3 physicians (RH.S, W.X and YD.F) were continuously included. Among them, malignant group (dysplasia/cancer) will be included until October 2020, and benign group will be included until 2020. The following patients were excluded: 1. Patients with diffuse gastric cancer; 2. Patients with GIM, but without specific subtype; 3. Patients with unclear final diagnosis; 4. Patients who suffered from any other malignant lesions except gastric mucosa tumors. Basic clinical data such as sex, age, smoking, alcohol, H pylori, bile reflux were collected.

### Test for *H. pylori*

The presence of *H. pylori* infection was determined using 13 C-urea breath test or the rapid urease test of gastric biopsy tissue. We considered the presence of *H. pylori* confirmed when either of these two tests were positive.

### Procedure of endoscopy

GIF-H260 (Olympus) was used in this study for endoscopic examinations. All endoscopic examinations were conducted by three independent physicians (RH.S, W.X, YD.F), who scanned the lesser curvature of the antrum (including angle) and the lesser curvature of the corpus using NBI endoscopy after a white light examination. At least one biopsy sample was then taken from the location that most likely to have GIM of these two areas respectively. A random biopsy was performed from the two areas respectively when physicians didn’t find any suspicious lesion. If there were other suspicious lesions, additional biopsies were performed. The most severe subtype of GIM of the same area was chosen to analyze.

### Histological assessment

All biopsies were separately fixed in buffered 10% formalin and embedded in paraffin. Paraffin-embedded samples were sliced and stained using eosin and hematoxylin (H&E), alcian blue, and the periodic acid Shiff reaction using the modified Giemsa method. Histological findings were recorded according to the updated Sydney System [18]**.** GIM subtype was then classified into complete and incomplete types. Another staining method was performed using high iron diamine (HID)-alcian blue for sulphomucin identification to distinguish type II from III GIM among the specimens with incomplete GIM.

### Scoring scheme

No GIM, type I, II, and III GIM were scored 0–3 points, respectively. The most severe subtypes of GIM of the gastric antrum (including gastric angle) and the gastric body were scored separately. The GIM severity of the whole stomach was calculated using two approaches. First, the gastric antrum and gastric body scores were added together, with a final score ranging from 0 to 6. This scoring system was named "Subtype Distribution Score of Gastric Intestinal Metaplasia (SDSGIM)". Second, direct classification according to “Table 1” which was similar to the method of OLGIM classification. The gold standard was the final pathological diagnosis, according to which the patients were divided into benign lesions and malignant lesions (dysplasia/cancer).

**Table 1:** Grading table of GIM based on the SDSGIM corresponding to table for OLGIM

Corpus	No IM (score 0)	Type I IM (score 1)	Type II IM (score 2)	Type III IM (score 3)
Antrum				
No IM (score 0)	Stage 0	Stage I	Stage II	Stage II
Type I IM (score 1)	Stage I	Stage I	Stage II	Stage III
Type II IM (score 2)	Stage II	Stage II	Stage III	Stage IV
Type III IM (score 3)	Stage III	Stage III	Stage IV	Stage IV

*SDSGIM* Subtype Distribution Score of Gastric Intestinal Metaplasia, *GIM* gastric intestinal metaplasia, *OLGIM* Operative Link on Gastric Intestinal Metaplasia

Stage III and IV were considered high-risk group

### Statistical analysis

SPSS version 18.0 (IBM Corp, Armonk, NY, USA) was used to analyze the date. Continuous variables were summarized as mean ± standard deviation and compared using Student's t test or Mann–Whitney U test. Categorical data were summarized using frequency tables. Univariate and Multivariate analysis was carried out to compare the impact of gender, age, *H. pylori* infection, smoking and drinking status, bile reflux, and SDSGIM scoring between benign lesions and malignant lesions (dysplasia/cancer). Receiver operating characteristic (ROC) curve was used to assess the diagnostic accuracy for dysplasia/cancer of SDSGIM scoring and to determine the optimal cut-off value. According to the optimal cut-off value and the classification data from Table 1, the patients were classified as high-risk (≥ cut-off value of SDSGIM; stage III/IV in Table 1.) and low risk (< cut-off value of SDSGIM; stage 0–II in Table 1) for malignancy. Using the pathological results as the gold standard, the sensitivity, specificity, likelihood ratios, and accuracy of these two classification methods for dysplasia/cancer were calculated. Furthermore, for patients with dysplasia/cancer, further comparisons were made between patients with multiple lesions and a single lesion using SDSGIM. The analysis was extended to evaluate the predicting value of SDSGIM for multiple malignant lesions. P value < 0.05 was considered to be statistically significant.

## Results

The study included 227 patients with an average age of 60.0 ± 10.9. The average age of patients with benign lesions, dysplasia, and cancer was found to increase along with the severity of diagnosis. There were 132 patients with benign lesions, 46 with dysplasia, and 49 with gastric cancer. All malignant lesions were pathologically diagnosed after ESD. Of the 227 patients, 100 were *H. pylori* positive (44.1%) and the average number of biopsies per person was 2.6 ± 0.7. For GIM, a total of 64 patients tested negative, 34 patients had GIM only in the gastric antrum, of which most (30/34) were in the benign lesion group. We found that 129 patients had GIM in the gastric corpus, and most of these patients (87/129) were with malignant lesions. Analysis of GIM subtypes of the whole stomach indicated that the majority of patients with type I GIM had benign lesions (23/28), while majority of patients with GIM subtype III had malignant lesions (57/72). The conditions of smoking, drinking, and bile reflux in each group were also shown in Table 2.

**Table 2:** Characteristics of 227 patients included

	Benign lesions	Dysplasia	Cancer	Total
Number of patients (%)	132 (58.1)	46 (20.3)	49 (21.6)	227 (100)
Sex (male/female)	71/61	41/5	43/6	155/72
Age (Mean ± SD)	57.9 ± 11.0	61.9 ± 9.2	63.7 ± 10.8	60.0 ± 10.9
*H. Pylori* (+) (%)	44 (44.0)	24 (24.0)	32 (32.0)	100 (100)
Smoking (+) (%)	22 (50.0)	8 (18.2)	14 (31.8)	44 (100)
Alcohol (+) (%)	20 (50.0)	11 (27.5)	9 (22.5)	40 (100)
Bile reflux (+) (%)	16 (51.6)	7 (22.6)	8 (25.8)	31 (100)
Biopsies (Mean ± SD)	2.5 ± 0.7	2.7 ± 0.7	2.6 ± 0.6	2.6 ± 0.7
Histology (%)				
Normal	60 (93.8)	3 (4.7)	1 (1.6)	64 (100)
Antrum IM	30 (88.2)	3 (8.8)	1 (2.9)	34 (100)
Corpus IM	42 (32.6)	40 (31.0)	47 (36.4)	129 (100)
Subtype of GIM (%)^a^				
Normal	60 (93.7)	3 (4.7)	1 (1.6)	64 (100)
Type I	23 (82.1)	2 (7.1)	3 (10.7)	28 (100)
Type II	34 (54.0)	17 (27.0)	12 (19.0)	63 (100)
Type III	15 (20.8)	24 (33.3)	33 (45.8)	72 (100)
SDSGIM				
Mean ± SD	1.4 ± 1.6	4.1 ± 1.5	4.5 ± 1.4	2.6 ± 2.1
Median and quartile	1 (0–2)	4 (4–5)	5 (4–5)	3 (0–5)

^a^The most severe subtype of whole stomach

**Fig. 1:**
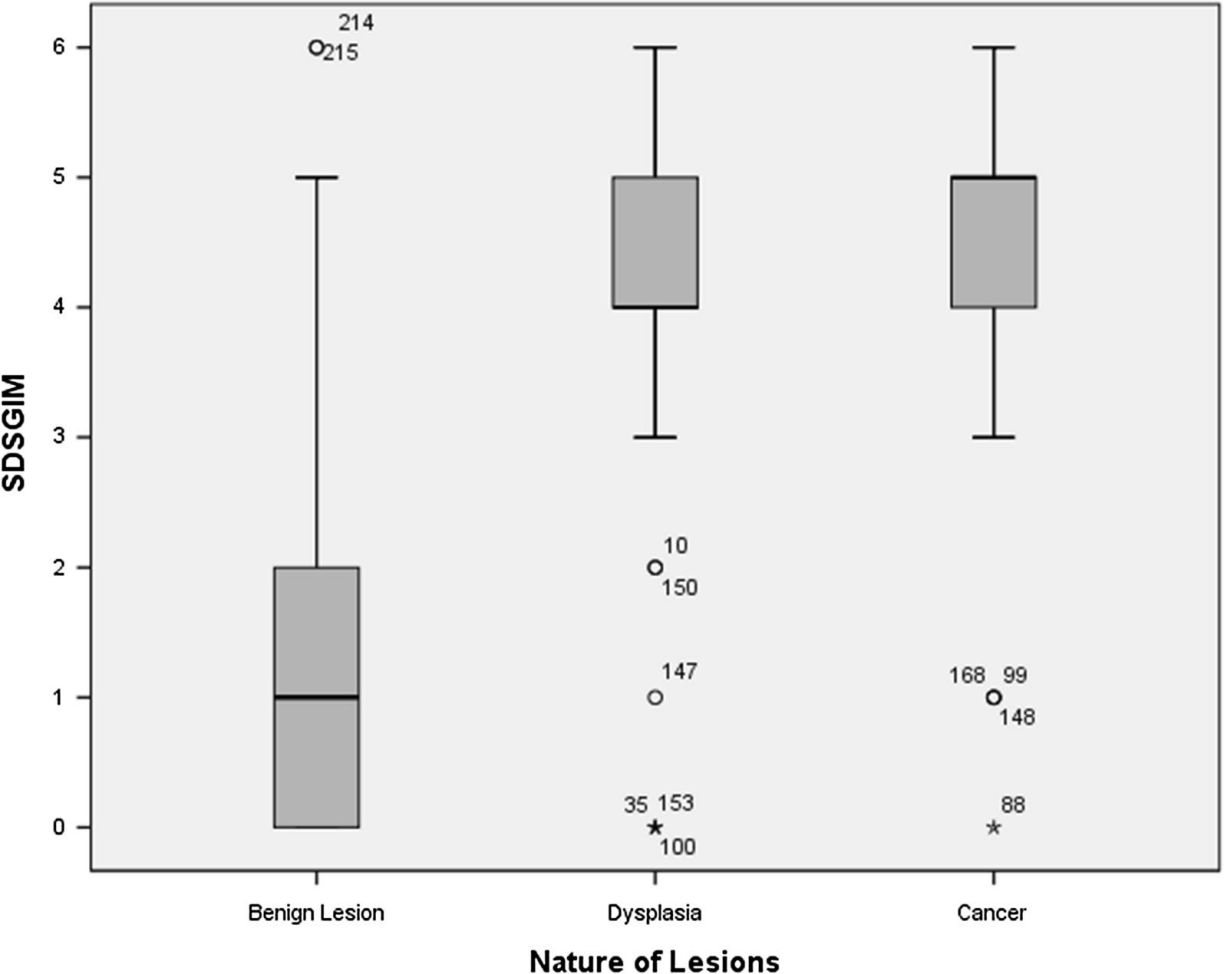
Subtype distribution score of gastric intestinal metaplasia and the nature of lesions, *SDSGIM* Subtype Distribution Score of Gastric Intestinal Metaplasia

For SDSGIM, benign lesion group (Mean ± SD: 1.4 ± 1.6) was significantly different from dysplasia (4.1 ± 1.5) (P < 0.001) and cancer group (4.5 ± 1.4) (P < 0.001). While there was no statistical difference between dysplasia and cancer group (P = 0.206) (Table2; Fig. 1). In order to exclude the influence of confounding factors, after dividing the lesions into benign and malignant groups (Dysplasia/Cancer), we further carried out a multivariate analysis. The results showed that the difference of SDSGIM between the two groups was still statistically significant (P < 0.001) (Table 3).

**Table 3:** Univariate and Multivariate analysis of related factors between benign and malignant (Dysplasia/Cancer) group

	Benign group	Malignant group	P value	
			Univariate analysis	Multivariate analysis
Number of patients (%)	132 (58.1)	95 (41.9)	–	–
Sex (male/female)	71/61	84/11	< 0.001	< 0.001
Age (Mean ± SD)	57.9 ± 11.0	62.8 ± 10.0	0.001	0.130
*H. Pylori* (+) (%)	44 (44.0)	56 (56.0)	< 0.001	< 0.001
Smoking (+) (%)	22 (50.0)	22 (50.0)	0.222	–
Alcohol (+) (%)	20 (50.0)	20 (50.0)	0.250	–
Bile reflux (+) (%)	16 (51.6)	15 (48.4)	0.427	–
SDSGIM			< 0.001	< 0.001
Mean ± SD	1.4 ± 1.6	4.3 ± 1.5	–	–
Median and quartile	1 (0–2)	5 (4–5)	–	–

**Fig. 2:**
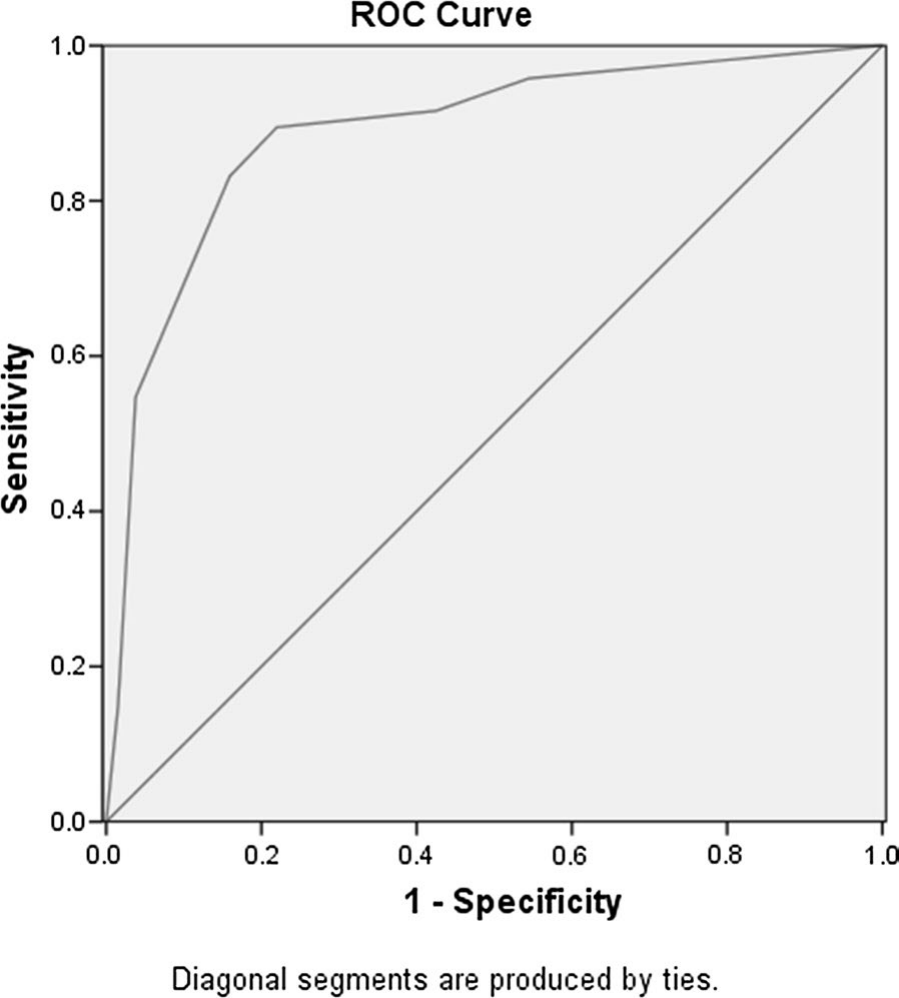
Receiver operating characteristic (ROC) curve of the SDSGIM for the nature of lesions

Receiver operating characteristic (ROC) curve of the SDSGIM lesion scoring data was drawn as shown in Fig. 2. The area under ROC curve (AUC) was 0.889 ± 0.023 (95%CI 0.843–0.934), showing that the used method had a good ability to distinguish benign from malignant lesions. According to the ROC curve data, the best cut-off value was estimated to be 3. Therefore, two groups were constructed using SDSGIM ≥ 3 for dysplasia/cancer group and SDSGIM < 3 for benign lesions groups. The sensitivity, specificity, positive predictive value (PPV), negative predictive value (NPV), and accuracy of SDSGIM scoring for malignancy were 89.5% (83.2–95.8), 78.0% (70.9–85.2), 74.6% (66.4–82.7), 91.2% (85.8–96.5) and 82.8% (77.9–87.8), respectively. And those for the high-risk group according to Table 1 (stage III or IV) was 84.2% (76.7–91.7), 82.6% (76.0–89.1), 77.7% (69.5–85.9), 87.9% (82.1–93.7), and 83.3% (78.4–88.2) respectively. (Tables 4, 5).

**Table 4:** The correlation between two methods of grading GIM and the pathology results (n)

Pathology results	SDSGIM^a^		Classification according to Table 1^b^	
	High-risk	Low-risk	High-risk	Low-risk
Dysplasia/Cancer	85 (41.9%)	10 (4.4%)	80 (35.2%)	15 (6.6%)
Benign lesions	29 (12.8%)	103 (45.4%)	23 (10.1%)	109 (48.0%)

*SDSGIM* Subtype Distribution Score of Gastric Intestinal Metaplasia

^a^High-Risk: SDSGIM ≥ 3; Low-Risk: SDSGIM < 3

^b^High-Risk: stage III or IV; Low-Risk: stage 0–II

**Table 5:** Predictive ability of two methods of grading GIM for malignant lesions (95%CI)

	Sensitivity	Specificity	PPV	NPV	Accuracy
SDSGIM	89.5% (83.2–95.8)	78.0% (70.9–85.2)	74.6% (66.4–82.7)	91.2% (85.8–96.5)	82.8% (77.9–87.8)
Table 1. Classification	84.2% (76.7–91.7)	82.6% (76.0–89.1)	77.7% (69.5–85.9)	87.9% (82.1–93.7)	83.3% (78.4–88.2)

*SDSGIM* Subtype Distribution Score of Gastric Intestinal Metaplasia, *PPV* positive predictive value, *NPV* negative predictive value

Area Under Curve was 0.889 ± 0.023 (95% CI 0.843–0.934). The optimal cut-off value should be 3.

We found that 95 patients had malignant lesions (SDSGIM score 4.33 ± 1.34), including 17 patients with multiple malignant lesions (SDSGIM score 4.33 ± 1.34) and 78 patients with single malignant lesions (SDSGIM score 4.12 ± 1.97). Nonparametric test results show that there was no significant difference in SDSGIM scores between patients with single and multiple malignant lesions (P = 0.583).

## Discussion

Hierarchical systems for GIM grading, including OLGIM and EGGIM, have several disadvantages including requirement of multi-point biopsies, poor objectivity, time-consuming protocol, and the necessity for highly trained specialists in endoscopy. Multiple studies have confirmed the value of GIM subtyping for predicting gastric cancer [15, 16]. A recent meta-analysis [17] showed that compared to complete GIM, incomplete GIM was associated with a 3.3-fold (95% CI, 1.96–5.64) increased risk of gastric cancer and 1.7-fold increased risk of progression to dysplasia. Compared with the severity of GIM that was used in the OLGIM system, subtype scoring is more commonly utilized in clinical pathology reports. In addition to subtypes, the distribution of intestinal metaplasia in gastric mucosa can also predict higher risk of progression to gastric cancer. GIM often originates in the lesser curvature of the gastric antrum and gradually spreads [19]. Compared with GIM detected solely in the gastric antrum, GIM in the stomach body may indicate a higher risk of malignant transformation [17]. The OLGIM system recommended five biopsies to grade the GIM. However, Meng Wang et al. [20] showed that biopsies from the lesser curvature of the gastric corpus, the angle, and the lesser curvature of the antrum could also accurately reflect the severity of GIM of whole stomach with fewer biopsy tissues. These results are similar to those found by Akiko Saka et al. [21]. Based on the above conclusions, we scanned the lesser curvature mucosa of the gastric antrum and corpus to evaluate the distribution and subtypes of GIM, establishing a novel GIM scoring system to provide a new method of GIM risk stratification.

The analyses of the 227 cases were consistent with previous studies. Gastric corpus GIM and type III GIM appeared more frequently in malignant lesions (dysplasia/cancer). After scoring the antrum and corpus separately, the two methods of GIM grading for malignant lesions—SDSGIM (cut-off was 3) and OLGIM risk stratification (Table 1; stage ≥ III)—both showed good prediction accuracy. In addition to intestinal subtype and GIM distribution, some factors such as smoking and drinking status, older age, gender, Bile reflux and *H. pylori* infection should also be considered for predicting the risk of gastric cancer. To exclude the confounding interference of these factors, a multivariate analysis was also performed. The analysis demonstrated a significant difference between scored benign and malignant lesions using SDSGIM method (P < 0.001). Furthermore, we explored the predictive value of SDSGIM scoring method for multiple malignant lesions. However, SDSGIM did not demonstrate predictive ability for malignancies with multiple lesions. Gisela Brito-Gonçalves et al. [22] reported a trend for patients with extensive GIM (EGGIM > 4) who had a higher risk of multiple lesions. The trend was not found significant using multivariate analysis. Suggestively, small sample size and the contingency of biopsy may be associated with negative predictive results and/or absence of statistical significance. Consequently, this conclusion requires further investigation.

This study also had some limitations. The chance of poor choice of biopsy sites may affect the scoring results, although the identification of GIM using NBI endoscopy [9] can help improving the stability of biopsy results. This study is a single-center small-sample study that decreases the power of the findings. Moreover, the source of the study objects was only inpatients which indicates the limited representation. Accordingly, our data warrants further prospective large-scale multi-center studies which should include outpatient sources. Besides, some important factors, including BMI and family history of cancer were not included in the analysis due to incomplete date, although some other confounding factors were included and analyzed using multivariate method. And this study could not compare the results of novel SDSGIM with OLGIM for lack of related date (OLGIM result) as a retrospective study. Finally, the rationality of the risk stratification OLGIM method (Table 1) requires further verification.

## Conclusions

This study firstly combined GIM subtypes with its distribution in gastric antrum and corpus to form a new scoring system, which showed high prediction accuracy for malignant lesions. The SDSGIM was significantly different between the benign lesions group and the dysplasia/cancer group.

## Supplementary Information

**Additional file 1.** Additional figure and tables.

**Additional file 2.** Original date.

## Data Availability

The datasets used and/or analyzed during the current study are available from the corresponding author on reasonable request.
