# Correction to: A novel method of grading gastric intestinal metaplasia based on the combination of subtype and distribution

**DOI:** 10.1186/s12935-021-01787-1

**Published:** 2021-02-24

**Authors:** Ning Wei, Zhiheng Zhong, Ruihua Shi

**Affiliations:** 1 Medical School of Southeast University, No. 87 Dingjiaqiao, Nanjing, 210009 China; 2 Department of Gastroenterology, Southeast University Affiliated Zhongda Hospital, No. 87 Dingjiaqiao, Nanjing, 210009 China

## Correction to: Cancer Cell Int (2021) 21:61 10.1186/s12935-021-01758-6

Following publication of the original article [1], we were notified that the corresponding author has been incorrectly flagged. The correspondence has been modified.

The original article has been corrected.
