# Supplementary material for: A novel method of grading gastric intestinal metaplasia based on the combination of subtype and distribution
Source: Cancer Cell Int. 2021 Jan 20;21:61. doi: 10.1186/s12935-021-01758-6 (PMC7816327; doi:10.1186/s12935-021-01758-6)
Supplement: Supplementary file 1 — Additional file 1. Additional figure and tables. [file 12935_2021_1758_MOESM1_ESM.docx]

**Table S1: Operative link on gastric intestinal metaplasia assessment (OLGIM) grading table.**

| **Corpus**  **Antrum** | **No** | **Mild** | **Moderate** | **Severe** |
| --- | --- | --- | --- | --- |
| **No** | 0 | I | II | II |
| **Mild** | I | I | II | **III** |
| **Moderate** | II | II | **III** | **IV** |
| **Severe** | **III** | **III** | **IV** | **IV** |

The figure below is quoted from Fig 3. of ”The Updated Sydney System”, which defines the severity described in Table S1.

**Figure S1:** The visual analog scale in the updated Sydney system.


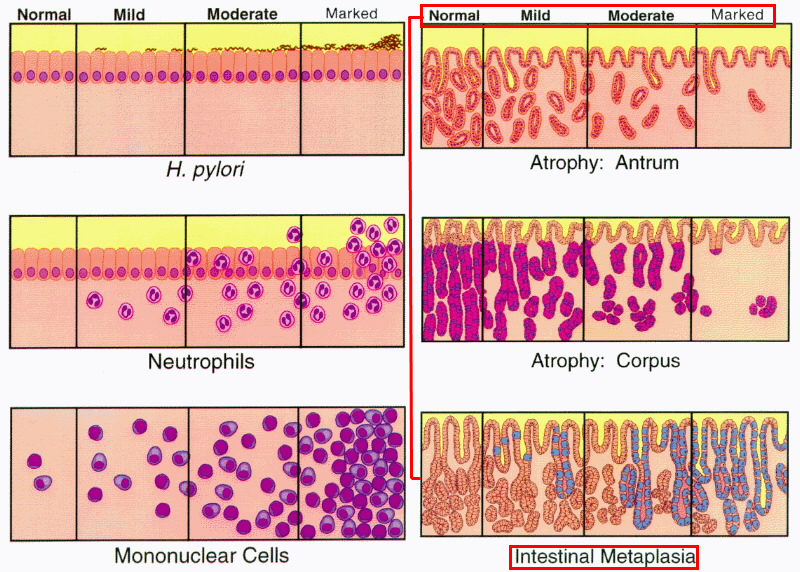


According to the percentage of cells of GIM under the microscope, the severity of GIM can be divided into four levels ( none, mild, moderate, and severe (or marked)).
